# Original Observations and Methods in the Practice of Medicine

**Published:** 1887-11

**Authors:** L. M. Stroud

**Affiliations:** Forney, Texas


### DANIEL'S TEXAS MEDICAL JOURNAL.

PUBLISHED MONTHLY.—SUBSCRIPTION $2.00 A YEAR.

Vol. 3. AUSTIN, NOVEMBER, 1887. No. 5.

### ORIGINAL ARTICLES.

CONTRIBUTED EXCLUSIVELY TO THIS JOURNAL.

*The Articles in this Department are accepted and published with the understanding that we are not responsible for, nor do we indorse the views and opinions of the writers by so doing.*

### ORIGINAL OBSERVATIONS AND METHODS IN THE PRACTICE OF MEDICINE.

*By L. M. Stroud, M. D., Forney, Texas.*

Read before the Terrell Medical Society, and, by vote, contributed to Daniel's Texas Medical Journal.

TO CORRECTLY estimate the therapeutic value of a drug in a particular disease, we must know how far to credit nature, and what would be the course of the disease uninfluenced by remedial agents.

A treatment, to be scientific, must be based on the pathology of the disease, and the physiological or chemical effects of the drug.

Many of us, doubtless, use some medicines empirically, because of their reputation in the pharmacopœa—never being able to perceive any effect, good or bad, of the remedies—the disease being self-limited, or having an intrinsic tendency to get well; in the course of years we acquire a vast accumulation of experience in favor of the drug, and possibly it becomes a specific and a hobby, and it is a notable fact, the more ignorant and unscientific the physician, the greater number of specifics he possesses.

And, Mr. President, I undertake to say, it is only by cultivating the boldest skepticism, as well as the keenest intuitive perception at the clinical bed, presupposing, of course, a thorough knowledge of the physiological functions and pathological conditions of the human body, that any of us can rise higher than a routine, stereotyped system of practice, becoming the mere apes of great original men in the profession.

It shall not be my purpose, at this time, to review the field of therapeutics, and pass upon the respective merits of each officinal drug; such an attempt would be an unwarrantable presumption in one of my age and clinical experience. However, I wish to direct attention to the value of certain methods of treatment, and the agents used, that I believe neither our text books nor the profession generally have fully acknowledged.

I will place Veratrum at the head of the list. Its best effect is seen in the treatment of pneumonia. I hold the great leading indication in the treatment of all acute inflammations is to lessen blood supply to the inflamed part. We accomplish this in a great variety of ways. We all appreciate the value of rest and position as influencing the circulation in inflammatory diseases. In an inflamed hand or foot, by position we lessen blood supply, and favor venous return, restoring the balance in the capillaries, between arterial supply and venous return; thereby relieving the distended capillaries, and securing resolution before effusion of lymph, or migration of white corpuscles has taken place. This latter result is always due to prolonged capillary congestion and over fullness, which has the effect to soften the walls of the capillaries, and so interfere with the nutrition of the inflammatory site as to produce the conditions of an abscess. If we can keep the circulation normal in an incised wound, it heals without inflammation, as is exemplified in union by first intention. I suppose you have all noticed that we never get union by first intention when there is much fever, or if the circulation is greatly disturbed. If the circulation is very active, blood is forced to the wound faster than it is returned from the capillaries, which causes migration of white corpuscles through the softened walls of the capillaries, and you get union by second intention.

Now, sir, pneumonia being inflammation of the lung, you do not want blood forced to the inflammatory area faster than the veins can return it from the capillaries. If we use a medicine in the first stage of this disease, we want something that will lessen the force and frequency of the heart's action. I have tried faithfully the list of cardiac and arterial sedatives for a number of years; veratrum is the only one that has never disappointed me; that has always brought the pulse down to its normal range, and held it there, with a corresponding fall of temperature.

For a number of years I indulged a prejudice against this remedy in pneumonia, believing that it could only weaken the heart that was already struggling against pulmonary obstruction, but by bringing the pulse down to its normal range, the veins are given time to collect and return the blood from the capillaries to the left auricle as fast as it is forced from the right ventricle to the lungs, and really a burden is taken off of the heart by lessening blood supply to the lungs; it also prevents further invasion of pulmonary area, by controlling active determination of blood to the lungs.

The heart is a muscle of wonderful reserve power, and will hold up till we get resolution, if it is not whipped by fever and inflammation beyond its speed and strength, while its right chambers are abnormally distended with venous blood trying to force blood into a solidified lung. We have all seen these cases die from failure of the heart about the ninth day, when we would get resolution in a short while, but for failure of the poor, over worked, exhausted heart.

Veratrum conserves the reserve power of the heart by not allowing it to become exhausted by excessive action. Veratrum has a powerful effect over the vaso-motor nervous system, contracting the arterioles, and lessening blood supply to the capillaries; this effect is produced in the inflamed lung as much as in other parts of the system. It has a sedative effect, also, over the pneumogastric, fainting, nervous cough which so greatly robs the patient of rest and sleep.

If any physician present is incredulous as to the power of veratrum to control inflammation, he can demonstrate its virtue for himself by healing his incised wounds by first intention, and aborting abscesses before the formation of pus. It may be proper just here to give my method of using it. It is such a powerful drug it is liable to great abuse:

We will suppose a case of pneumonia in the first stage. As soon as I am called, I administer a hypodermic injection of Norwood's tr. veratrum, about six or seven drops, combined with a little hydro chlorate of cocaine, to prevent pain, and repeat half the amount every half hour till patient vomits, which is the physiological effect of the drug, giving copious draughts of hot water to protect the stomach during vomiting. After this, two or three drops, given every three hours by the mouth, will be sufficient to control the circulation twenty-four hours; but I generally press it to emesis at the beginning of each respiral exascerbation.

To sum up the effects of veratrum, as I have been able to confirm at the clinical bed :

1. By holding the pulse between 65 and 70, it prevents further invasion of pulmonary area; and if your patient has breathing space enough to live with, he will not die from increased dyspnœa.

2. The patient will not die from failure of the heart, as 70 pulsations to the minute will not exhaust its reserve power.

3. The patient will not die from purulent infiltration of the lung as this is the result of a heart beating from a hundred, to a hundred and twenty to the minute, pumping more blood to the inflamed lung than the veins can return; keeping the capillaries distended so long their walls soften and break down.

4. It will not paralyze the heart nor general circulation of a weak patient, as I reduced the pulse of a feeble little girl fron 120 to 75 pulsations to the minute, it being a case of pneumonia with tardy resolution, and as late as the fourteenth day the patient was breathing forty to the minute, and of a feeble constitution, and it was evident her reserve power was nearly exhausted. She was kept under the influence of the drug two days, when the breathing became natural, and she made a good recovery. The patient, and the physician with whom I was in consultation, both live in this county, and I may mention, by way of parenthesis, the Doctor is a convert to veratrum.

Next, I wish to attest the value of sulphuric ether in congestions. We never hear of a death from nervous shock, or failure of the circulation on the surgeon's table, where ether is used during the operation. The surgeon knows its value, but I do not believe the general practitioner fully appreciates the range of its application. It matters little what form of congestion—whether it be a congestive chill, general capillary congestion, cholera morbus, cholera infantum, malarial capillary congestion of infants producing tonic convulsions, so you cannot prize the jaws open to give a dose of medicine, or whether it be incipient dysentery—if you will place the patient on the table, and give ether to the surgical degree, the patient will become very red, the pulse will have a good volume, and instead of congestion in some organ, or in the general capillary circulation, you will have the blood circulating uniformally in the skin, extremities and entire capillary system; and in the event of tonic convulsions of infants, where the patient generally dies as much from asphyxia as from congestion, which is due to the respiratory muscles being in a state of prolonged tonic spasm, rendering breathing impossible, your patient will become relaxed, and thoroughly under the influence of the ether in three minutes, if you will fill the cone with ether, and perform artificial respiration vigorously. As soon as the patient becomes red, the breathing becomes soft and easy. In any case of congestion, the patient should be kept under the influence of ether till other appropriate remedies have time to take permanent effect.

By this method of treatment you will generally abort most diseases that begin as congestions.

Next, I wish to speak of the use of chloroform in cerebro-spinal meningitis. A case in point of illustration: Mr. A. S. Thompson, a young man 27 years of age, was taken with a chill, and in a short time was seized with a convulsion, his head and heels being nearly drawn together in the opisthotonon position. The trismus was so great his jaws could not be prized open to give a dose of medicine. The convulsion was so long and violent, it appeared he would die from asphyxia, as breathing was impossible. The patient finally relaxed, and bromide of potassium and calomel were given in large doses; but shortly the second convulsion came on, and, if possible, was more violent than the first. This finally passed off, and shortly, symptoms of the third convulsion began to appear. The patient would partially recover intellection after each convulsion. He now began to beg most pitifully for assistance, saying if he had the third spasm he would die. The patient was thoroughly relaxed with chloroform, and the impending attack was averted. A trusted nurse was placed by his side, with orders to give chloroform till patient was thoroughly relaxed, when patient was threatened with recurrence of convulsions. Patient had symptoms of convulsions for two days, but was kept relaxed with chloroform. In the meantime, patient was saturated with bromide of potassium, calomel and quinine. Made a good recovery, except a lameness in left leg for life.

Next, I wish to mention a method of treating catarrhal state of the bladder. When the mucous membrane of this viscus is once in a state of chronic inflammation, it is generally a source of annoyance to the physician, and of extreme discomfort to the patient. While opiates quiet irritability, and the urine can be made bland by neutralizing its uric acid, yet the patient remains uncured, and as the last of the urine is voided, the patient suffers pain, and a scalding sensation as the bladder is trying to empty itself of the irritating mucus that has settled to the most dependent part of the bladder—the mucus being too thick and tenacious to pass through the urethra, it remains in the bladder to decompose, and become more acrid, and to add to former accumulations.

The method of treatment I wish to mention, is simply distending, and washing out the bladder by means of a fountain syringe with a soft rubber catheter attached. Injections into the bladder amount to very little, unless it is distended, so as to put on the stretch all the corrugated folds of mucous membrane, and dislodge all mucous accumulations. This can be very ingeniously done by first injecting a weak solution of cocaine into the bladder through the soft rubber catheter, before attaching syphon of syringe. This will prevent spasm of the bladder during the operation. Then fill the fountain of syringe with warm water representing the capacity of the distended bladder. You can place fountain of syringe at any desired height. When all the water is in the bladder, disconnect the catheter from syphon of syringe, and allow everything to wash out through the catheter. Then repeat the operation with a two per cent. solution of sulphate of zinc, so as to completely cut all the mucus out of the corrugated folds of mucous membrane, and again wash out through the catheter. A few applications of this kind, if the patient is kept quiet and warm, and under the influence of remedies to make his urine bland, will suffice for a complete cure. I think the most grateful patient I ever had, was a lady who had suffered for more than a year with catarrh of the bladder, who was cured completely by one application. Of course, constitutional conditions should be looked after, such as the uric acid diathesis, etc.

Next, I wish to mention a method of treating a case of placenta prævia. Case: Mrs. N. was taken in labor, at full term, about a year ago. Had been having ante-partem hemorrhages for two weeks previous—at one time hemorrhage was profuse. The physician in attendance found her exsanguined, and fainting from loss of blood, with hemorrhage still pouring from her. There was no probability of labor terminating itself speedily, as the os was only dilated the size of half a dollar, and pains were feeble and irregular. I was sent for, and reached the case in a few minutes. Found her fainting every few minutes, and blood still pouring from her. The physician in attendance had apprised her husband of impending death, and had written a telegram to be sent to her mother in a distant State, that she was dying. Her countenance had the extreme palor of death; breathing was shallow and irregular.

On examination, I found the placenta presenting, and removed a small bit of it to be certain of my diagnosis. The Doctor thought it useless to try to do anything further. However, we quickly agreed to try to deliver her, if she died while we were making the attempt. We raised her bed so as to make her heels about three feet higher than her body. The heart was so feeble, this stopped the hemorrhage completely. I introduced the largest size Barnes' dilator. Dilation was easy and rapid, owing to the relaxed condition of patient. The dilator brought on efficient pains. I removed the placenta and performed version. I suppose we completed the dilation, version and delivery in ten minutes. Contraction was good, and patient made a good recovery.

I forgot to say, fainting ceased as soon as we increased the elevation of toot of the bed, which threw all the blood she had to the heart and brain, blood supply to these centers of life being a condition of the performance of their vital functions.

I believe it possible to save the mother in most cases of placenta prævia by means of gravitation, Barnes' dilators, and version.

Now, Mr. President, I wish to make an observation on the abuse of quinine in typhoid fever, at the close of this paper. Quinine has a powerful effect over the vaso-motor nervous system, and predisposes to hemorrhage. You can demonstrate this by giving a lady a large dose of quinine during menstruation. In the majority of instances it will excite sufficient hemorrhage to place her in bed. I have a case in private practice, in which a single dose of quinine produces hæmaturia. In a case of purpura hæmorrhagica associated with epistaxis, every time I gave a dose of quinine it excited hemorrhage. I am satisfied I have seen more than one case of hemorrhage of the bowels in typhoid fever induced by large doses of quinine.

It may be insisted that hemorrhage in typhoid fever is due to a dyscrasia of the blood and a loss of tone in the vaso-motor nervous system. A predisposition to hemorrhage only enforces the reason why quinine should be thought dangerous.

A liability to excite hemorrhage is not the only objection to quinine in typhoid fever. It irritates the stomach, and causes the patient to loathe food, often exciting vomiting, and completely interfering with nutrition, which is sheet anchor in the treatment.

There is yet another objection to quinine in this fever. It is a great irritant to an ulcer. You can demonstrate this by bathing a slight sore on your hand in a solution of quinine. You will scarcely be able to heal it while the application is continued. This serves to illustrate the effect of quinine on the ulcerated Peyer's patches, inflaming and deepening the ulcers, to say nothing of the danger of exciting hemorrhage from that locality.

The best authors do not claim that quinine will either abort or abridge the duration of typhoid fever. In my opinion, its antipyretic effect is more than counterbalanced by the inconvenience and dangers of its use, as we have much more reliable and less dangerous agents that will control temperature. The germ of typhoid fever is of animal origin, and is not destroyed by quinine, as is its cousin, the bacillus malariæ, which is of vegetable parentage. However, in a malarial district, if I believed the patient impressed with malaria, I would give it, sufficient to overcome periodicity, and then discontinue its use.

